# Investigation of Calcium Forms in Lichens from Travertine Sites

**DOI:** 10.3390/plants11050620

**Published:** 2022-02-25

**Authors:** Dajana Ručová, Tamara Đorđević, Matej Baláž, Marieluise Weidinger, Ingeborg Lang, Andrej Gajdoš, Michal Goga

**Affiliations:** 1 Department of Botany, Institute of Biology and Ecology, Faculty of Science, Pavol Jozef Šafárik University, Mánesova 23, 041 67 Košice, Slovakia; dajana.rucova@upjs.sk; 2 Department of Mineralogy and Crystallography, University of Vienna, Althanstrasse 14, 1090 Vienna, Austria; tamara.djordjevic@univie.ac.at; 3 Department of Mechanochemistry, Institute of Geotechnics, Slovak Academy of Sciences, Watsonova 45, 040 01 Košice, Slovakia; balazm@saske.sk; 4 Core Facility Cell Imaging and Ultrastructure Research, University of Vienna, Djerassiplatz 1, 1030 Vienna, Austria; marieluise.weidinger@univie.ac.at; 5 Department of Functional and Evolutionary Ecology, University of Vienna, Djerassiplatz 1, 1030 Vienna, Austria; ingeborg.lang@univie.ac.at; 6 Institute of Mathematics, Faculty of Science, Pavol Jozef Šafárik University, Jesenná 5, 040 01 Košice, Slovakia; andrej.gajdos@upjs.sk

**Keywords:** calcium oxalate, X-ray diffraction, Raman spectroscopy, EDX analysis, lichens

## Abstract

Lichens are symbiotic organisms with an extraordinary capability to colonise areas of extreme climate and heavily contaminated sites, such as metal-rich habitats. Lichens have developed several mechanisms to overcome the toxicity of metals, including the ability to bind metal cations to extracellular sites of symbiotic partners and to subsequently form oxalates. Calcium is an essential alkaline earth element that is important in various cell processes. Calcium can serve as a metal ligand but can be toxic at elevated concentrations. This study investigated calcium-rich and calcium-poor sites and the lichen species that inhabit them (*Cladonia* sp.). The calcium content of these lichen species were analyzed, along with localized calcium oxalate formed in thalli collected from each site. The highest concentration of calcium was found in the lichen squamules, which can serve as a final deposit for detoxification. Interestingly, the highest content of calcium in *Cladonia furcata* was localized to the upper part of the thallus, which is the youngest. The produced calcium oxalates were species-specific. Whewellite (CaC_2_O_4_∙H_2_O) was formed in the case of *C. furcata* and weddellite (CaC_2_O_4_∙2H_2_O) was identified in *C. foliacea*.

## 1. Introduction

Lichens primarily obtain mineral nutrients from atmospheric deposition [1]. The soil is another important source of mineral nutrients. In general, essential nutrients are classified as macroelements and microelements, according to their concentration in lichens or plant tissue [2,3]. Calcium (Ca) belongs to the group of macroelements and is relatively immobile in most plant species [4]. This essential element participates in various metabolic and physiological reactions [5]. Ca is an important structural component of cell walls as well as biological membranes. Further functions of this divalent cation are related to its ability to modify the permeability of cell membrane. Ca also plays a key role in the phosphorylation of nuclear proteins, cell division, protein synthesis, and carbohydrate metabolism as well as in water and solute movement [6]. In addition, Ca acts as a molecular messenger in the cytosol [5,7]. In plants, Ca is required in responses to biotic or abiotic stress and hormonal signals [5]. In lichens, we assume a comparable role of Ca for homeostasis as in other organisms. Because Ca is an important ecological factor for species distribution in lichens, this study distinguishes calciphile species from calciphobe species even though the physiological role of Ca deficiency or excess in lichens is largely unknown [6].

Lichens inhabit various substrates, including those with high content of Ca or another metals. Ca is absorbed from the mineral substrate and then either precipitated in intracellular compartments or extracellularly secreted in a form of CaOX (calcium oxalate) deposits. Lichen production of CaOX is a very carefully regulated process due to the secretion of oxalic acid and the formation of calcium in a form that is non-toxic, stable, and non-diffusible [8]. In areas with highly abundant soluble Ca, a regulation mechanism for excess Ca is necessary. One of the physiological or biochemical options is the production of osmotically inactive CaOX crystals [9].

Crystals of CaOX may have different morphologies in lichens. Whewellite (monohydrate) usually forms styloids, flat hexagons, cubes, and multifaceted conglomerate crystals (druses). The most common morphologies of weddellite (dihydrate) are bipyramids, bipyramidal prisms, and druses [10]. Weddellite may change its mineral form to whewellite due to considerably lower thermodynamic stability [11]. The formation of specific shapes and sizes of oxalate crystals is genetically regulated and indicated by the coordination of crystal growth within lichen species [9].

The most common types of Ca biominerals are CaOX crystals. The first report about CaOX crystals in plants dates back to the late 1600s, when Leeuwenhoek described these objects under his light microscope. CaOX is an insoluble organic salt [12] that can be found in microorganisms, algae, plants, and animals. Lichen thalli may contain large amounts of Ca ions [13]. The CaOX in these organisms is produced by the reaction of oxalic acid and Ca acquired from the environment. Oxalic acid (C_2_H_2_O_4_) is a biologically synthesized byproduct of the fungal lichen partner. While it is one of the simplest carboxylic acids, it is strong and the most highly oxidized organic acid. Oxalic acid is soluble in water and acts as a chelator of metal ions [14]. The oxalates it acquires are closely related to the chemical composition of the rock on which lichens grow [14].

Although three types of CaOX minerals can occur in nature, just two of them are found in lichens: the monohydrate whewellite, CaC_2_O_4_∙H_2_O (crystallizing monoclinic, space group *P*2_1_/*c*) [15] and the dihydrate weddellite CaC_2_O_4_∙(2 + x)H_2_O (crystallizing tetragonal, space group *I*4/*m*) [15,16,17]. Recent work [18,19] showed a significant variation in the amount of “zeolite” H_2_O molecules and a positive correlation between these and several crystal chemical parameters, including the unit cell parameters.

CaOX crystals are mostly found in lichens growing on limestone [20], serpentinite [21], and sandstone [22]. In the study of limestone [20], the production of a certain amount of CaOX is a characteristic feature of calcicolous lichens. Interestingly, CaOX can also be found in lichens grown on travertine sites. The terms travertine and tufa (sometimes used synonymously) refer to all non-marine carbonate precipitates. These form near lakes, rivers, and caves, but mainly near or directly at terrestrial springs [23]. Many biotic and abiotic factors influence travertine formation [24]. The term travertine is associated with Ca carbonate deposits made by non-marine, supersaturated Ca bicarbonate-rich waters [25].

In the present study, we investigated the potential accumulation of excess Ca from travertine sites in different parts of lichen thalli. For comparison, the Ca content in lichens from calcium-poor areas were analyzed too. Raman spectroscopy and X-ray diffraction were used for identification of CaOX forms.

## 2. Results

### Elemental Analysis (EDX) of Studied Lichen Species

**Figure 1:**
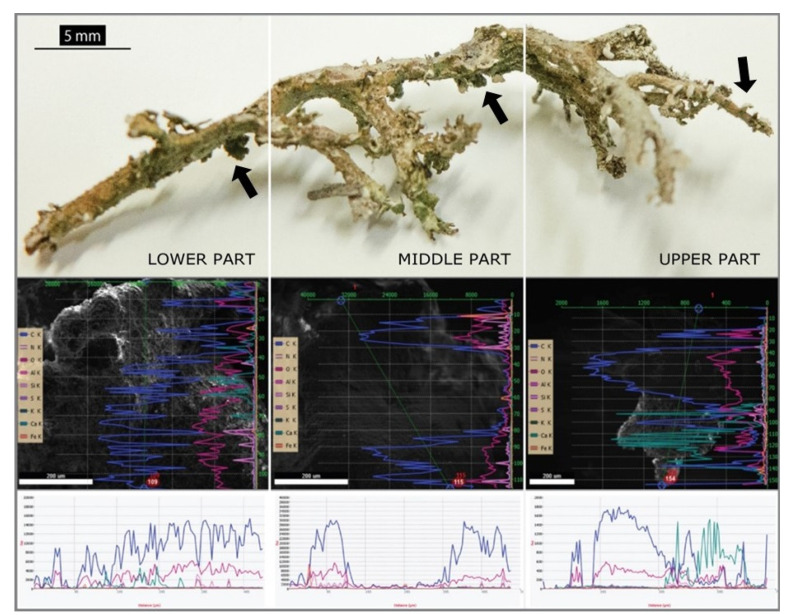
Line scans of three selected parts of lichen *Cladonia furcata* (turquoise color represents Ca), where the highest amount was measured in the upper part of the lichen thallus. Black arrows indicate squamules.

**Figure 2:**
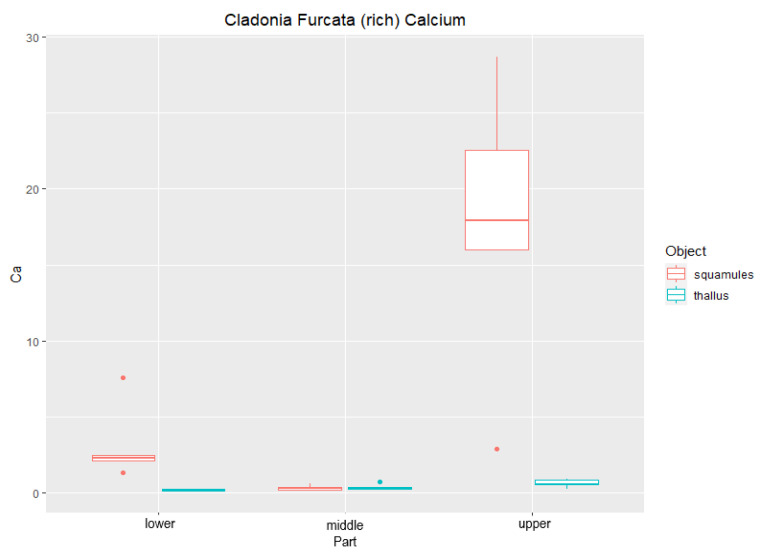
Content of Ca (DW%) in lichen thallus and squamules of *Cladonia furcata* collected from calcium-rich sites (*n* = 5). “Lower”, “middle”, and “upper” designate the location of the measured area.

**Figure 3:**
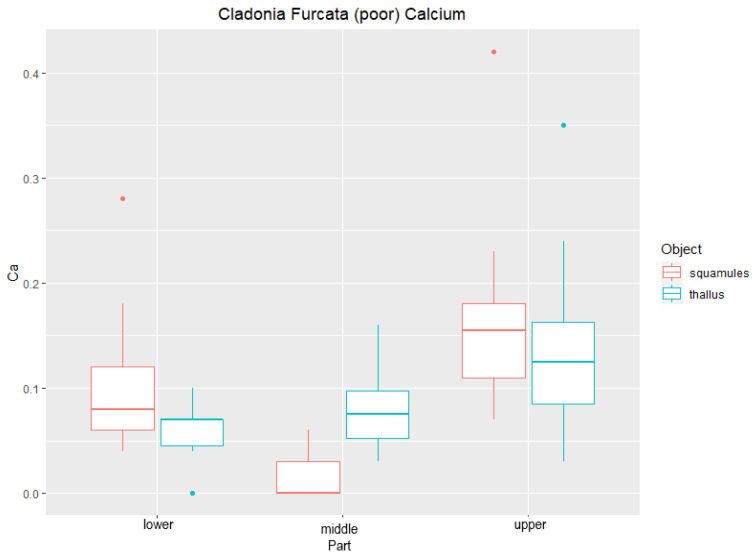
Content of Ca (DW%) in lichen thallus and squamules of *Cladonia furcata* collected from calcium-poor sites (*n* = 5). “Lower”, “middle”, and “upper” designate the location of the measured area.

Line scans of *C. furcata* from all three parts of lichen thalli were analyzed horizontally. Measurements showed the concentration of calcium to be higher in the upper part than in the lower part (Figure 1). The amount of Ca was significantly higher in the squamules as well, which rise from the sides of lichen thalli. This can be seen in (Figure 2), where the average content of Ca was 0.58% in the upper thallus and 17.61% in the squamules (DW). Significantly different content of Ca was measured in thallus and squamules from Ca-poor sites (Figure 3), where the average of values was 0.14% from the upper part of the thallus and 0.17% from squamules (DW).

**Figure 4:**
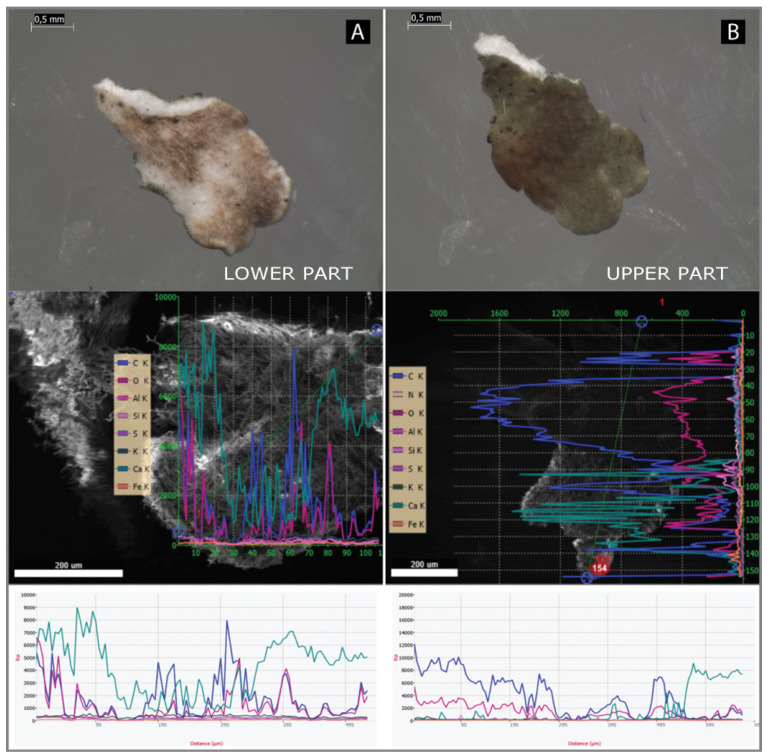
(**A**) Line scan of *Cladonia foliacea* thallus, measured from the lower part; (**B**) line scan of *Cladonia foliacea* thallus, measured from the upper part. Turquoise color represents Ca, with the highest amount measured in the lower part of the lichen thallus.

**Figure 5:**
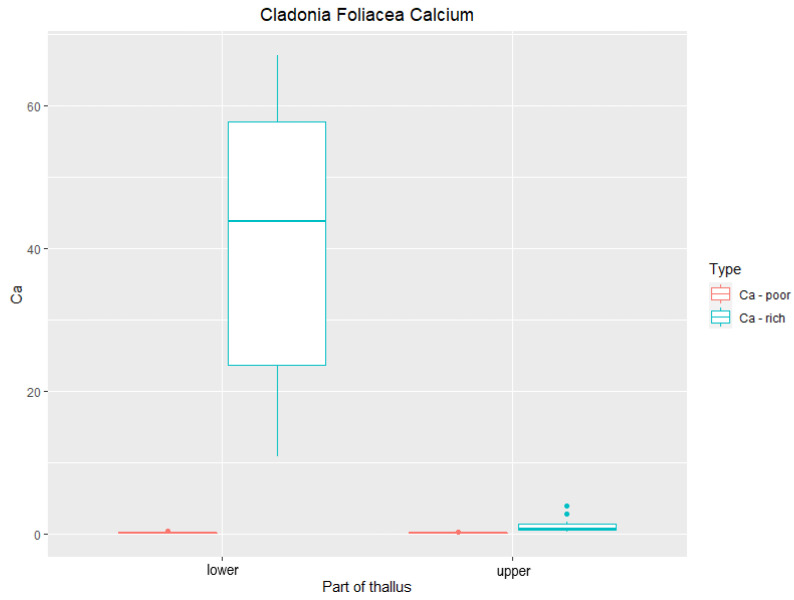
Content of Ca (DW%) in lichen *Cladonia foliacea* collected from calcium-rich and calcium-poor sites (*n* = 5). “Upper” and “lower” designate the location of the measured area.

In the species *C. foliacea*, the line scan (Figure 4) measurement showed a different phenomenon. Its squamulose primary thallus only consists of a lower part and upper part, with the highest concentration of Ca cumulated in lower part of the squamulous thallus. According to Figure 5, the average of Ca in the upper part was 1.187%, whereas it was 41.75% (DW) in the lower part. The content of Ca in *C. foliacea* thallus from Ca poor-sites was 0.1% (DW) in the upper part, whereas it was 0.13% in the lower part (DW) (Figure 5).

Line scans for elemental analyses were measured transversely along the podetium with the squamules (Figure 1). The highest amount of Ca was measured in the upper part, especially on the squamules. In contrast to *C. furcata*, *C. foliacea* has a dorsiventral thallus. We analyzed the lower part (close to the substrate) and the upper part (photosynthetically active layer). Line scans for elemental analyses were measured transversely on the lobe (Figure 4A). In *C. foliacea,* the highest content of Ca was on the lower part of the lichen thallus.

**Figure 6:**
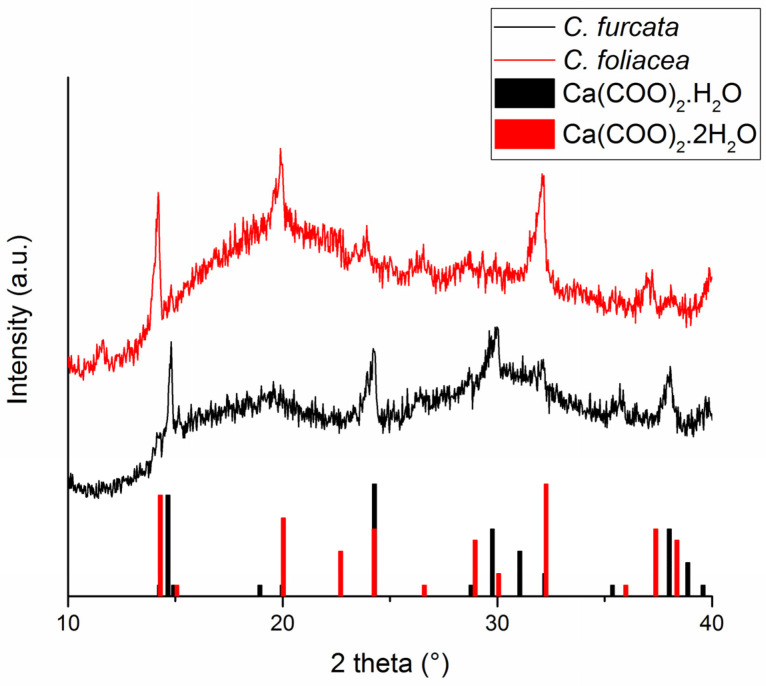
XRD pattern of the Ca-containing *C. furcata* (**black pattern**) and *C. foliacea* (**red pattern**) and corresponding intensities of the identified CaOX species according to JCPDS-PDF2 database (black line—35-0914, red lines—04-0702). The corresponding phases and formulas are provided in the figure.

To prove and determine the presence of CaOX, PXRD patterns were measured (Figure 6). According to the PXRD, CaOX found are whewellite (calcium oxalate monohydrate) and weddellite (calcium oxalate dihydrate). In the case of C. *furcata*, monohydrate modification, i.e., whewellite, was observed. Dihydrate modification (weddellite) was found for *C. foliacea*.

**Figure 7:**
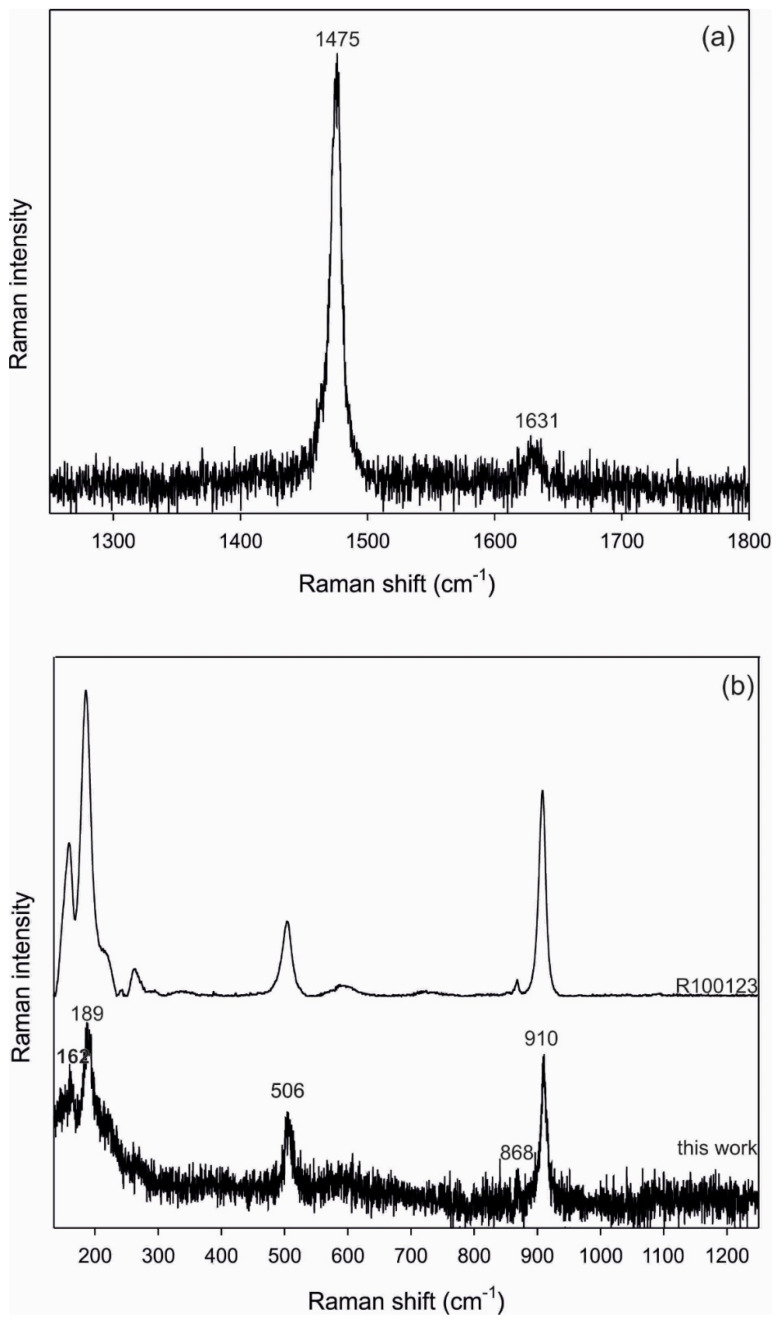
Raman spectrum of weddellite (**a**) in the C-C stretching region and (**b**) in the range from 100 to 1300 cm^-1^ (the comparison to spectrum of weddellite sample R100123 from RRUFF database (rruff.org) is also provided).

**Figure 8:**
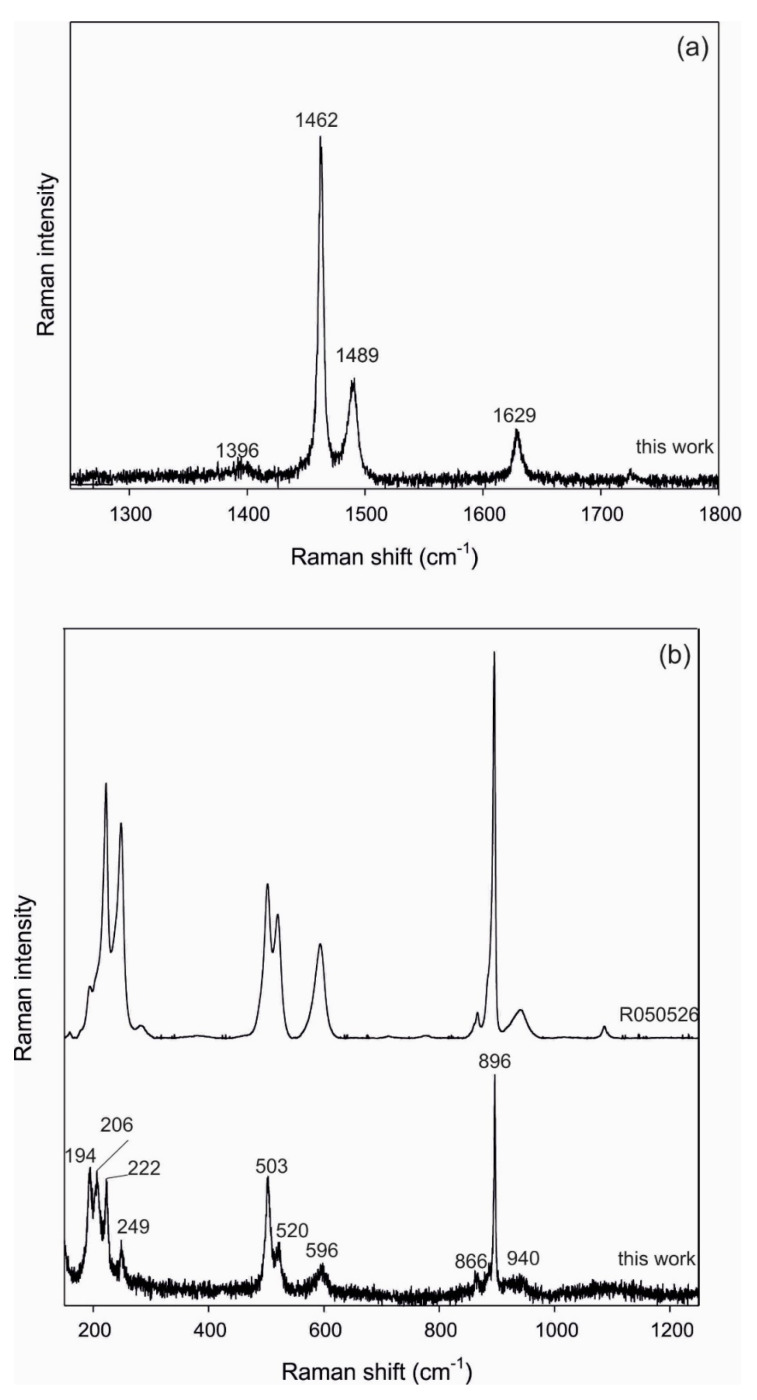
Raman spectrum of whewellite (**a**) in the C-C stretching region and (**b**) the range from 100 to 1300 cm^-1^ (the comparison to spectrum of whewellite sample R050526 from RRUFF database (rruff.org) is also provided).

Thus, both CaOX hydrate minerals, weddellite (CaC_2_O_4_∙2H_2_O) in *C. foliacea* (Figure 7) and whewellite (CaC_2_O_4_∙H_2_O) in *C. furcata* (Figure 8), were identified in the lichens by Raman spectroscopy. The Raman spectra of the CO stretching in the region 1250–1800 cm^−1^ are shown in (Figure 7) and (Figure 8). The bands identified in the 1450–1500 cm^−1^ range are assigned to the *ν*(C–O) stretching modes.

## 3. Discussion

At the ecosystem level, lichens may be considered one of the organisms that are most vulnerable to pollution [26]. As a result, lichens are widely used for biomonitoring atmospheric pollutants such as acid rain, nitrogen compounds, heavy metals, and calcium [27]. Polluted environment can have a significant impact not only on mankind but also on another living systems including lichens [28,29,30]. Because of their ability to accumulate certain elements, there is increased interest in the potential of lichen to act as pollution monitors and important regulators of mineral-cycling processes [31]. Previously, sensitivity and Ca-tolerance have been documented in terricolous and epilithic lichens [32]. The influence that lichens can have on Calcium-rich areas may thus be related to their ability to bind Ca in the form of CaOX.

Crystalline deposits corresponding to CaOXs are mainly present on the upper surface of the lichen body or on internal fungal hyphae of the lichen thallus [33]. Fundamentally, lichens obtain Ca from two sources: air and substrate [6].

In our experiment with *C. furcata*, the highest content of Ca was measured in the upper part, the most metabolically active and youngest part of the lichen thallus. It is known that the upper part of this fruticose thallus is branched, resulting in a larger area of exposure. *C. furcata* is a podetia lichen form with an inconspicuous primary thallus. The surface of this lichen is very smooth and occasionally highly squamulose. The habitat of *C. furcata* is usually on open soil, but other habitats include shaded grasslands, lowland heaths, roadsides or rock outcrops, and forests [34]. The highest amount of Ca in *C. furcata* was in the upper and lower parts, which could be influenced by the above-mentioned facts. On the other hand, *C. foliacea,* with a different growth form, showed the highest accumulation of Ca on the lower part. This is often close to the substrate.

Deposits of CaOX, generally known as pruina [17] or oxalate pruinosity [35] are present on the surface of some lichen thalli from habitats with high calcium levels. In the lichens investigated, the formation of whewellite and weddellite can be explained by the significant concentration of Ca ions from travertine substrate.

Using RS, it is possible to discriminate between hydration states of CaOX, weddellite (1473 and 1631 cm^−1^) and whewellite (1392, 1462, 1489, and 1629 cm^−1^). The measured values correlate well with the literature data [36,37,38] for both minerals. It is substantial that whewellite, which is monohydrate, was determined in fruticose lichen *C. furcata* and weddellite, which is dihydrate, was found in foliose lichen *C. foliacea*. We assume that this phenomenon is related to the differing anatomies of lichens. Weddelite serve for absorption and accumulation of water. If the humidity in the environment decreases, this changes to whewellite [39,40]. *C. furcata,* as podetia lichen forms, are exposed to the open environment, which also influences evaporation. There are not many opportunities to create a micro-environment. *C. foliacea,* as squamulose lichen forms, have better opportunities to create micro-environments which better preserve the humidity related to the substrate.

Positions and relative intensities of the Raman bands for the C–C stretching region (800–1100 cm^−1^) and the low-frequency region are shown in Figure 7 and Figure 8. An intense Raman band is observed at 910 cm^−1^ for weddellite and is assigned to the *ν*(C–C) stretching mode. For the whewellite, this band appears at 896 cm^−1^ as a single intense band. The low intensity band around 866 cm^−1^ in both spectra is assigned to the OCO bending mode [36]. In the low-frequency region, the measured intensities of weddellite (163, 189 and 506 cm^−1^) and whewellite (194, 206, 222, 249, 503, 520, and 596 cm^−1^) reflect the major bands of the corresponding compounds reported in the literature [36,37,38]. The results of Raman spectroscopy are thus in accordance with the X-ray diffractions.

The lichen primary thallus of *Cladonia* species contains squamules. In some species, squamules are lost during ontogenesis of the thallus, e.g., in the subgenus *Cladina*. Among other functions, squamules are mainly used for asexual reproduction. They are easily broken off the main thallus and may be distributed by animal activity, trampling, or wind, before generating new thalli. The podetia are a secondary thallus in *Cladonia* species, which are overlaid with squamules. *C. foliacea* has a squamulose-to-foliose primary thallus. It can be found on open soil that is often calcareous and acidic. Sand dunes and rock outcrops are additional habitats where this lichen can be found [34]. Another potential role of squamules, as a detoxification structure in Ca excess, has been considered in our findings.

Giordani et al. [8] observed the monohydrated whewellite and the dihydrated weddellite in 43 lichen specimens from an herbarium, including 17 pruinose foliose thalli. In this experimental study, only pruinose lichens that produced CaOXs aggregates on their upper cortex were selected. According to this study, we took lichens from their natural habitats to test the possibility of CaOX production. Due to the fact that investigated lichen species grow in habitats with elevated amounts of Ca, they regulate the excess amount of Ca by the formation of CaOX crystals. However, the production of CaOX was shown to be absent in the older parts of the thallus as well as in cortical hyphal cells [11]. Active extension via growth of foliose lichens occurs only at the edges of the lobes [41]. Furthermore, CaOX formation appears to play a crucial role in other important functions. Its main purposes are the regulation of Ca levels; detoxification; protection from herbivory; Ca storage; light gathering; and reflection [11,42,43]. While these functions have been described for higher plants, evidence remains lacking for many of these functions in lichens.

## 4. Material and Methods

### 4.1. Collection of Material and Study Areas

Lichen material was collected from travertine sites in autumn (October 2017) in Slovakia. Bešeňová travertine (49°06′23″ S 19°26′15″ W) was chosen for lichen collection. *Cladonia furcata* (Hudson) and *Cladonia foliacea* (Hudson) from this locality were identified by Michal Goga and confirmed by Anna Guttová. Lichen specimens (*Cladonia furcata* KO36523), (*Cladonia foliacea* KO36522) from this locality are deposited in the Botanical Garden of University of Pavol Jozef Šafárik. Control of lichen thalli *C. foliacea* was collected in the lowland steppe, open, sandy, perennial grassland (Pannonian psammophytic grasslands) area Tece of Vácrátót [44]. Voucher specimens (VBI-L 6104, 6105) are deposited in the VBI Lichen Herbarium (Vácrátót, Hungary). Control thalli of (*C. furcata* KO36524) was collected in Zemplínske vrchy from reddish sandstone with rhyolite.

### 4.2. Elemental Analysis (EDX) with a Scanning Electron Microscope (SEM)

The podetia of *C. furcata* are secondary parts of the thallus that look like upright stems. Based on the age and fruticose growth type, we divided the thallus into the lower (oldest), middle (intermediate), and upper (youngest) parts. All three of these parts contain thallus and squamules. Elemental analysis of selected elements from our samples was performed using a JEOL JSM IT 300 scanning electron microscope (SEM) equipped with an EDAX system (Ametek GmbH, Weiterstadt, Germany) for energy-dispersive X-ray microanalysis (EDX). Lichen material was mounted on 0.5″ aluminium specimen stubs covered with SEM-carbon foils (PELCO TabsTM Carbon Conductive Tabs, Double Coated, Christine Gröpl, Tulln an der Donau, Austria). Samples for EDX were dried and carbon-coated with a 5–10 nm carbon layer (Carbon coater, MED 020; Leica, Wetzlar, Germany) to prevent surface charge. For specific spectra analyses, background subtraction, and data collection, the EDAX-TEAM software Version V4.3 (Ametek Material Analysis, Mahwah, NJ, USA) was used. For deconvolution of the spectra, corrections for interference between elements were applied according to the software. Elemental analyses were performed with the following constant SEM settings: acceleration voltage of 20 kV; working distance 11 mm (sample to final lens); take-off angle 35.1; dead time 30%; and measurement time 50 s (Lsec 50) for each measurement. From each, sample multiple measurements (*n* = 5–10) were taken. Considering that samples show uneven surface and texture, all elemental analyses were performed at a magnification of 850×. Attention was taken that the orientation of the sample surface with respect to the Silicium Drift Detector (SDD), model Octane Plus Det, geometry was kept stable. This minimized the chance of a possible blur of the measurements. Ca concentrations are shown as weight percentages of the dry mass (DW%).

### 4.3. Powder X-ray Diffraction

The samples selected for powder X-ray diffraction (PXRD) were homogenized and powdered by fine grinding in an agate mortar. Homogeneous powders of all four samples were analyzed using a D8 Advance X-ray diffractometer (Bruker, Billerica, MA, USA) in the Bragg-Brentano geometry, working with a CuKα (λ = 0.15418 nm) radiation and a scintillation detector. The operating voltage and current were 40 kV and 40 mA, respectively. All samples were scanned from 10° to 40° with steps 0.03° and 15 s counting time. The DIFFRAC.EVA software and the ICDD powder diffraction file PDF-2 were used for peak and phase identification.

### 4.4. Raman Spectroscopy

Natural Ca oxalate hydrate minerals are most often identified using Raman spectroscopy. They are usually recognized by the Raman position of the CO stretching vibration, which is cation-sensitive. These bands are observed at around 1460 cm^−1^ and around 1630 cm^−1^. To obtain further information on the anion groups, especially on the C–O stretching vibrations and on the hydrogen bonds, Raman spectra of weddelite and whewellite were acquired. They were measured with a Horiba LabRam–HR system equipped with an Olympus BX41 optical microscope in the spectral range between 100 and 4000 cm^−1^. The 632.8 nm excitation line of a He–Ne laser (10 mW) was focused with an Olympus 100× objective (N.A. = 0.90) on the randomly oriented single crystals. The spectra were acquired with a nominal exposure time between 10 and 20 s (confocal mode, 1800 grooves/mm grating in the beam path, 1.5 µm lateral resolution and approximately 3 μm depth resolution).

### 4.5. Statistics

We employed a two-way ANOVA approach to compare the amount of calcium in *C. foliacea*, both rich and poor types, in the lower part versus the upper part. This methodology was also helpful in the case of *C. furcata* (rich and poor separately), when investigating the influence of two factors and their interaction on the amount of calcium. Namely, we examined the influence of the the object type (thallus versus squamules) and the influence of the part type (upper part, middle part, and lower part) of *C. furcata*. Multiple comparisons were made by using Tukey Honest Significant Difference (HSD) procedure.

Statistical analysis was conducted in open source statistical software/language R (in Integrated Development Environment RStudio) [45,46]. We used the following R packages: readxl—to read data from MS Excel; stats—to compute ANOVA; Tukey HSD; and ggplot2—for visualizations.

## 5. Conclusions

Metal binding with organic acids, oxalate crystals, lichen secondary compounds, polysaccharides, and pigments are well-known detoxification mechanisms. Here, the formation of different calcium oxalate minerals as a regulation mechanism for excess calcium has been observed in both categories of tested lichens. For these lichen species, squamulous formation is a natural phenomenon. It seems that the detoxification of excessive amounts of calcium takes place in squamules, which enable these species of lichens to grow on a substrate rich in calcium as well. Furthermore, though squamules have been used mainly as a taxonomic sign, we report a potential role of squamules in the detoxification mechanism. Further studies are required to determine which other roles may exist.

## Data Availability

The data can be provided on reasonable request by the authors.
